# Effects of Single‐Session Treatment of Primary Teeth Under General Anesthesia Versus At‐Office Multi‐session Treatment on Permanent Molar Caries Status: Nonrandomized Clinical Trial

**DOI:** 10.1002/cre2.70187

**Published:** 2025-08-28

**Authors:** Shirin Taravati, Sahar Tehrani, Masoumeh Khataminia, Vahid Rakhshan

**Affiliations:** ^1^ Department of Pediatric Dentistry, School of Dentistry Ahvaz Jundishapur University of Medical Sciences Ahvaz Iran; ^2^ Formerly, Department of Dental Anatomy Azad University of Medical Sciences Tehran Iran

**Keywords:** caries formation, dmft (decayed, missing, and filled teeth index of primary teeth), general anesthesia, International Caries Detection and Assessment System (ICDAS), OHI‐S index (simplified oral hygiene index), oral hygiene, pediatric dentistry, permanent dentition, primary dentition, tooth decay

## Abstract

**Objectives:**

Effects of treatment under general anesthesia versus several sessions of at‐office treatment on the transition of caries prevalence from primary to permanent dentitions have not been assessed. Moreover, other gaps exist in the literature.

**Material and Methods:**

This 4‐group non‐randomized clinical trial was performed on 280 children 8–10 years old in 4 groups, including both negative and positive controls: children with a history of caries in primary teeth who were (1) treated routinely in office (*n* = 60) versus (2) under general anesthesia versus (*n* = 110) (3) similar children undergoing no treatment versus (positive control, *n* = 60) (4) children without a history of primary teeth caries (negative caries, *n* = 50). A clinical assessment was performed to assess dmft (caries and restoration statuses of primary teeth), OHI‐S (oral hygiene), and ICDAS indexes (caries and restoration statuses of permanent molars). Data were analyzed (α = 0.05).

**Results:**

There were significant, positive correlations among dmft, caries component of ICDAS, and OHI‐S (*p*‐values = 0.000). The dmft scores were significantly different across 4 groups (Kruskal–Wallis *p* = 0.000), with children treated under general anesthesia followed by in‐office treatment and no‐treatment positive control group having the highest dmft scores (all Bonferroni‐adjusted *p*‐values ≤ 0.013). The ICDAS‐caries of the general anesthesia group was significantly greater than the negative control, but also significantly smaller than both positive control and in‐office treatment group (all Bonferroni‐adjusted *p*‐values ≤ 0.009). Groups were different in terms of OHI‐S (Kruskal–Wallis *p* = 0.000); hygiene was the worst in positive‐control and in‐office groups, followed by the anesthesia group and the negative control.

**Conclusions:**

Although children who underwent treatment with general anesthesia had the worst dmft scores, treatment under general anesthesia considerably reduced caries of their permanent first molars (as indicated by ICDAS scores) and oral hygiene (as indicated by OHI‐S) compared to children who were treated routinely in the office. Primary tooth caries might be a decisive predictor of permanent molar caries formation.

## 1 Introduction

One of the most common chronic diseases in children all over the world is tooth decay, which is also an infectious and multifactorial disease (McDonald et al. 2011; Khosravi et al. 2021). Dental caries is extremely important, especially in the case of the first permanent molars, because they are key to mastication, occlusion, maintaining vertical height, and facial form. Therefore, their early loss causes many problems, such as increased overbite, reduced mastication, among others (Antonsen and Hunstad 2011).

Primary teeth play an important role in children's healthy nutrition, speaking, and appearance as well as in the growth of healthy permanent teeth; therefore, the importance of these teeth should not be neglected (Crawford and Lennon 1992; Grytten et al. 1988). Decay of these teeth may lead to serious problems such as pain and swelling, among others (Crawford and Lennon 1992). Moreover, there is a strong association between the molar caries and the previous decays in deciduous teeth; for example, the decay on the distal surface of the deciduous second molar tooth at the age of 6–12 years affects the mesial surface of the permanent first molar tooth (Mejàre and Stenlund 2000). Due to incomplete primary enamel mineralization or hypoplastic defects during tooth eruption, the first molars' calcification process is completed during the first 2 years after eruption by exposure to saliva (Seraj 2002; Heymann et al. 2012). The maximum incidence of new caries lesions occurs in the first year after their eruption (Antonsen and Hunstad 2011). Considering that the permanent first molar is the first tooth that grows in the mouth at the age of 6–7 years (and has a long growth period), it is the most susceptible tooth to decay (Antonsen and Hunstad 2011).

So far, few studies have investigated the effect of the history of caries of primary teeth on the caries of the permanent first molar (Jafari et al. 2021; Gormley et al. 2023; Lam et al. 2022). In addition, the status of primary teeth and oral hygiene in patients whose first permanent molars required pulp treatment before the age of 10 years has not been investigated before. Especially, when it comes to comparatively evaluating the role of treatment under general anesthesia versus office treatment in such a link, it is even more scarce. Treatment under general anesthesia may improve the quality of life of children and their health as well as their or their parents' motivation to improve their oral hygiene (Novaes et al. 2017; Gupta and Raghu 2021; Razeghi et al. 2020). We aimed to compare this treatment option with treatment in the office because the treatment in the office is done over a long period of time, and it is possible to teach dental hygiene and follow up on the given training. The null hypotheses were a lack of association between any of the independent variables (prior primary dentition status, treatments received, types of treatments, sex, and age) on dependent variables (caries formation in maxillary and mandibular permanent molars on the left and right sides).

## 2 Materials and Methods

This prospective non‐randomized clinical trial was performed in 2023–2024 in Ahvaz, Iran. The protocol and its ethics were approved by the Medical Ethics Committee of Ahvaz Jundishapur University of Medical Sciences, Ahvaz, Iran (ethical code: IR.AJUMS. REC.1402.309). Written informed consent was obtained from all parents. The data were confidential.

### 2.1 Sample Size

A sample size of 4 groups of 30 each (a total of 120 individuals) was adequate to obtain powers above 80% at a 0.05 level of significance, based on results of a previous study (Songur et al. 2019) (*s* = 1.89 and *d* = 1.4). We increased the sample size to 280 patients. All groups surpassed the minimum required size of 30 children per group.

### 2.2 Sample

All procedures (all dental treatments and also all diagnostic procedures) were performed by the same experienced pediatric dentist. The inclusion criteria were systemically normal and healthy children without any systemic diseases or syndromes in the age range of 8‐10 years who were willing to cooperate in the study and whose parents agreed and signed written informed consent forms. The study comprised 4 groups:

1. The **general anesthesia** group: children in the age range of 8–10 years old with a history of caries in primary teeth who were treated in one session with general anesthesia before the eruption of permanent first molar teeth (*n* = 60 children). The reason for selecting this treatment setting was merely the clinical indications and clinical limitations judged by an experienced pediatric dentist in accordance with textbooks. The treatment of choice was always the in‐office treatment unless it was not justifiable or practical. For instance, very uncooperative and very young children with several severe caries might be an indication for one‐session treatment under general anesthesia – as long as there was no contraindication present.
2. The **in‐office** group: children in the age range of 8–10 years old with a history of primary tooth caries who have undergone routine dental treatment in the office before the eruption of permanent first molars (*n* = 110). This treatment setting was always the treatment of choice, unless not possible due to clinical limitations, where general anesthesia would be considered.
3. The **positive control** group: 8 to 10‐year‐old children with a history of caries in primary teeth before the eruption of permanent first molars and no treatment until after the eruption of permanent first molars (*n* = 60).
4. The **negative control** group: ‎8‐ to 10‐year‐old children with a history of no caries in primary dentition, who were considered as the negative control group (*n* = 50). The condition of their permanent dentition was not a criterion for their enrollment.

### 2.3 Assessments

Each of the children was examined by an experienced pediatric dentist (the only examiner) with a dental mirror and a probe under the light of the dental unit. The following indices were calculated:

*‎–‎ dmft*

The status of primary teeth of all groups was determined using the dmft index (decayed, missing, and filled teeth index of primary teeth, Table S1; Meamar et al. 2000).

*‎–‎ OHI‐S*

The oral hygiene status of the samples was also determined according to the OHI‐S index (simplified oral hygiene index, Table S1) (Sosiawan et al. 2022).

*‎–‎ ICDAS index*

After determining the OHI‐S index, the state of health or decay of the first molar teeth is examined. We used the International Caries Detection and Assessment System (ICDAS) for this purpose. Using this index, all stages of enamel and dentin decay as well as tooth restoration can be investigated and recorded. This index is a composite score consisting of 2 digits. The first digit (the left one) indicates the restoration status of the surfaces, and the second digit indicates the decay status of the surfaces (Table S1) (Scully 2013).

### 2.4 Statistical Analysis

The ICDAS system uses a composite score consisting of 2 different categories (left digit for restorations and right digit for caries). Therefore, it was converted into two scores: ICDAS – caries and ICDAS – restorations. Each of these components was analyzed separately. Descriptive statistics and 95% confidence intervals (CI, where applicable) were computed for the variables in different groups and sexes. An independent‐samples *t*‐test was used to compare the ages in boys and girls. A one‐way analysis of variance (ANOVA) was used to compare the ages in the 4 groups. A chi‐square test was used to compare sex distributions in the 4 groups. A chi‐square test was used to compare the 4 study groups in terms of distributions of d, m, f, and dmft scores across the 4 groups as well as the ICDAS scores for restoration and caries statuses for each of the 4 molars as well as the OHI‐S scores for each of the 6 teeth, and finally the categorical overall OHI‐S status (poor, average, good determined based on the thresholds stated above). A Kruskal–Wallis and a Bonferroni‐adjusted post‐hoc test were used to compare the 4 study groups in terms of their dmft scores as well as the average of the right digits (caries status) in their ICDAS scores, and their average OHI‐S scores. A Mann–Whitney *U* test was used to compare boys and girls in terms of their dmft scores, the average of the right digits (caries status) in their ICDAS scores, and their average OHI‐S scores. A Spearman correlation coefficient was used to examine the correlations among dmft, ICDAS ‎–‎ caries, OHI‐S, and age. The software in use was SPSS 27 (IBM, Armonk, NY, USA). The level of significance was set at 0.05.

## 3 Results

There was no missing data. There were 280 children with a mean age of 8.97 ± 0.809 years (95% CI: 8.82–9.11). These included 157 girls and 123 boys. The mean age of girls was 9.05 ± 0.799 years (95% CI: 8.92–9.18). In boys, the average age was 8.97 ± 0.809 years (95% CI: 8.82–9.11). In each sex, the minimum and maximum ages were 8 and 10, respectively. There was not a significant difference between the average ages of boys and girls (*t*‐test, *p* = 0.389). Moreover, the average ages of children in the 4 groups were not significantly different (one‐way ANOVA, *p* = 0.466, Table 1). There were 50 children in the “healthy” group (negative control, 31 girls, 19 boys), 60 children in the “no treatment” group (positive control, 40 girls, 20 boys), 110 children in the “office treatment” group (58 girls, 52 boys), and 60 children in the group “one‐session treatment under general anesthesia” (28 girls, 32 boys). The distributions of males and females were not significantly different between the 4 groups (chi‐square, *p* = 0.107). Descriptive statistics for dmft, ICDAS – caries component (the average of ICDAS – caries component for all the 4 molar teeth in each child), and OHI‐S indexes are presented in Table 2.

**Table 1:** Descriptive statistics and 95% CIs for age in different groups. In each of the 4 groups, the minimum and maximum ages were 8 and 10 years, respectively.

Group	*N*	Mean	SD	95% CI	
Healthy	50	9.10	0.789	8.88	9.32
No treatment	60	8.97	0.823	8.75	9.18
Office	110	8.95	0.799	8.79	9.10
Anesthesia	60	9.12	0.804	8.91	9.32

Abbreviations: CI, confidence interval; SD, standard deviation.

**Table 2:** Descriptive statistics for dmft, ICDAS – caries component (the average of 4 molar teeth per child), and OHI‐S indexes in different study groups.

Group	Parameter	*N*	Mean	SD	Min	Max	Q1	Med	Q3
Healthy (negative control)	dmft	50	0.000	0.000	0.000	0.000	0.000	0.000	0.000
	ICDAS caries	50	0.115	0.221	0.000	1.000	0.000	0.000	0.250
	OHI‐S	50	0.617	0.432	0.000	1.667	0.333	0.667	0.875
No treatment (positive control)	dmft	60	5.900	2.927	1.000	12.000	4.000	5.000	8.000
	ICDAS caries	60	2.371	1.496	0.000	6.000	1.250	2.000	3.188
	OHI‐S	60	1.250	0.517	0.333	2.500	0.833	1.083	1.500
Office	dmft	110	7.573	1.742	2.000	12.000	7.000	8.000	8.000
	ICDAS caries	110	2.184	1.794	0.000	6.000	0.500	2.000	3.563
	OHI‐S	110	1.053	0.439	0.000	2.333	0.792	1.000	1.333
General anesthesia	dmft	60	9.400	1.942	6.000	14.000	8.000	10.000	11.000
	ICDAS caries	60	0.775	1.109	0.000	5.750	0.000	0.500	1.000
	OHI‐S	60	0.942	0.447	0.000	2.000	0.667	0.833	1.333

Abbreviations: Max, maximum; Med, median; Min, minimum; Q1, first quartile; Q3, third quartile; SD, standard deviation;

### 3.1 Correlations

According to the Spearman correlation coefficient, there were positive and statistically significant correlations among all 3 parameters: dmft, OHI‐S, and the average of ICDAS – caries component for all 4 first molar teeth (Table 3). Age was not correlated with any of the 3 parameters (Table 3).

**Table 3:** The results of the Spearman correlation coefficient (Rho). *N* = 280 for each of the correlation coefficients.

		ICDAS – caries (average)	dmft	OHI‐S
Age	Rho	−0.081	−0.006	−0.081
	*p*	< 0.001	0.924	0.176
ICDAS – caries (average)	Rho		0.331	0.360
	*p*		< 0.001	< 0.001
dmft	Rho			0.243
	*p*			< 0.001

### 3.2 dmft

The Kruskal–Wallis test indicated a significant difference among the dmft scores in the 4 groups (*p* = 0.000, Figure S1). The Bonferroni‐adjusted post hoc test showed 6 significant pairwise comparisons between each two groups (all 6 Bonferroni‐adjusted *p*‐values ≤ 0.013). The chi‐square test showed significant associations between “treatment types” (the 4 groups) with each of the items: d, m, f, and dmf (Table 4). The Mann–Whitney *U* test showed a nonsignificant (marginally significant) difference between dmft scores of boys and girls (*p* = 0.084, Figure S2).

**Table 4:** Contingency tables showing the net distributions (and percentages) of various dmf scores of primary teeth (as in decay, missing, and filling) across different treatment groups.

		Treatment groups (%)				
Parameter	Score	Healthy	No treatment	Office	Anesthesia	*p*
Decay (d of dmft)	0	50 (31.1)	2 (1.2)	58 (36)	51 (31.7)	< 0.000001
	1	0	3 (20)	6 (40)	6 (40)	
	2	0	11 (33.3)	21 (63.6)	1 (3)	
	3	0	3 (27.3)	8 (72.7)	0	
	4	0	18 (66.7)	8 (29.6)	1 (3.7)	
	5	0	6 (66.7)	3 (33.3)	0	
	6	0	5 (45.5)	5 (45.5)	1 (9.1)	
	7	0	2 (66.7)	1 (33.3)	0	
	8	0	3 (100)	0	0	
	9	0	1 (100)	0	0	
	10	0	5 (100)	0	0	
	12	0	1 (100)	0	0	
Missing teeth (m of dmft)	0	50 (27.6)	37 (20.4)	62 (34.3)	32 (17.7)	0.000014
	1	0	5 (11.4)	20 (45.5)	19 (43.2)	
	2	0	4 (16.7)	13 (54.2)	7 (29.2)	
	3	0	2 (33.3)	3 (50)	1 (16.7)	
	4	0	8 (47.1)	8 (47.1)	1 (5.9)	
	5	0	0	1 (100)	0	
	6	0	2 (66.7)	1 (33.3)	0	
	8	0	2 (50)	2 (50)	0	
Filling (f of dmft)	0	50 (40.3)	60 (48.4)	14 (11.3)	0	< 0.000001
	1	0	0	2 (100)	0	
	2	0	0	8 (100)	0	
	3	0	0	8 (88.9)	1 (11.1)	
	4	0	0	12 (85.7)	2 (14.3)	
	5	0	0	9 (69.2)	4 (30.8)	
	6	0	0	14 (73.7)	5 (26.3)	
	7	0	0	7 (58.3)	5 (41.7)	
	8	0	0	32 (62.7)	19 (37.3)	
	9	0	0	1 (33.3)	2 (66.7)	
	10	0	0	3 (23.1)	10 (76.9)	
	11	0	0	0	4 (100)	
	12	0	0	0	8 (100)	
dmft (d + m + f)	0	50 (100)	0	0	0	< 0.000001
	1	0	2 (100)	0	0	
	2	0	5 (71.4)	2 (28.6)	0	
	3	0	2 (66.7)	1 (33.3)	0	
	4	0	15 (78.9)	4 (21.1)	0	
	5	0	8 (61.5)	5 (38.5)	0	
	6	0	9 (37.5)	11 (45.8)	4 (16.7)	
	7	0	1 (6.3)	12 (75)	3 (18.8)	
	8	0	6 (7.1)	59 (69.4)	20 (23.5)	
	9	0	1 (16.7)	3 (50)	2 (33.3)	
	10	0	7 (22.6)	9 (29)	15 (48.4)	
	11	0	0	2 (28.6)	5 (71.4)	
	12	0	4 (28.6)	2 (14.3)	8 (57.1)	
	13	0	0	0	2 (100)	
	14	0	0	0	1 (100)	

*Note:* The *p*‐values are calculated using the chi‐square test.

### 3.3 ICDAS

There was no tooth with both ICDAS components being a number greater than zero at the same time; in other words, if there was any form of restoration (left digit > 0), no caries was detected (right digit = 0), and vice versa. The chi‐square test indicated significant associations between “treatment types” (the 4 groups) with each of ICDAS scores (both digits) (Table 5). The average score for the right digit of ICDAS (caries status) of the 4 molar teeth was calculated for each person. The Kruskal–Wallis test showed a significant difference among the 4 groups in terms of their average ICDAS‐caries scores (*p* = 0.000, Figure S3). The Bonferroni‐adjusted post hoc test showed 5 significant pairwise comparisons (all 5 Bonferroni‐adjusted *p*‐values ≤ 0.009) except the groups positive control and office treatment, which did not significantly differ from each other (*p* = 0.739, Figure S3). The Mann–Whitney *U* test did not detect a significant difference between average ICDAS‐caries scores of boys and girls (*p* = 0.575, Figure S4). None of the ICDAS scores 96, 97, 98, 99 (reserved for missing teeth) were observed in this study.

**Table 5:** Contingency tables for the net distributions (and percentages) of various ICDAS scores (indicative of various degrees of decay and different types of dental restorations) across different treatment groups.

		Treatment groups (%)				
Parameter	Score	Healthy	No treatment	Office	Anesthesia	*p*
ICDAS UR molar restoration (left digit)	No restoration	50 (18.5)	57 (21.1)	109 (40.4)	54 (20)	0.048
	Complete sealant‎	0	0	0	3 (100)	
	amalgam‎	0	2 (40)	1 (20)	2 (40)	
	SSC	0	1 (50)	0	1 (50)	
ICDAS UL molar restoration (left digit)	No restoration	50 (18.5)	57 (21)	110 (40.6)	54 (19.9)	0.021
	Complete sealant‎	0	0	0	3 (100)	
	Amalgam‎	0	2 (50)	0	2 (50)	
	SSC	0	1 (50)	0	1 (50)	
ICDAS LR molar restoration (left digit)	No restoration	50 (19.5)	54 (21)	109 (42.4)	44 (17.1)	< 0.000001
	Complete sealant‎	0	1 (10)	0	9 (90)	
	Amalgam‎	0	2 (25)	1 (12.5)	5 (62.5)	
	SSC	0	3 (60)	0	2 (40)	
ICDAS LL molar restoration (left digit)	No restoration	50 (19.2)	56 (21.5)	109 (41.9)	45 (17.3)	< 0.000001
	Complete sealant‎	0	0	0	9 (100)	
	Amalgam‎	0	0	1 (20)	4 (80)	
	SSC	0	4 (66.7)	0	2 (33.3)	
ICDAS UR molar caries (right digit)	Intact	49 (31.2)	15 (9.6)	47 (29.9)	46 (29.3)	< 0.000001
	ICDAS 1	1 (2.2)	16 (35.6)	20 (44.4)	8 (17.8)	
	ICDAS 2	0	15 (60)	9 (36)	1 (4)	
	ICDAS 3	0	2 (22.2)	4 (44.4)	3 (33.3)	
	ICDAS 4	0	6 (20.7)	22 (75.9)	1 (3.4)	
	ICDAS 5	0	1 (16.7)	5 (83.3)	0	
	ICDAS 6	0	5 (55.6)	3 (33.3)	1 (11.1)	
ICDAS UL molar caries (right digit)	Intact	49 (32.2)	19 (12.5)	41 (27)	43 (28.3)	< 0.000001
	ICDAS 1	1 (2.2)	15 (33.3)	21 (46.7)	8 (17.8)	
	ICDAS 2	0	13 (50)	11 (42.3)	2 (7.7)	
	ICDAS 3	0	3 (33.3)	4 (44.4)	2 (22.2)	
	ICDAS 4	0	5 (16.7)	21 (70)	4 (13.3)	
	ICDAS 5	0	1 (20)	4 (80)	0	
	ICDAS 6	0	4 (30.8)	8 (61.5)	1 (7.7)	
ICDAS LR molar caries (right digit)	Intact	39 (35.8)	7 (6.4)	30 (27.5)	33 (30.3)	< 0.000001
	ICDAS 1	11 (21.6)	11 (21.6)	16 (31.4)	13 (25.5)	
	ICDAS 2	0	14 (36.8)	15 (39.5)	9 (23.7)	
	ICDAS 3	0	7 (70)	3 (30)	0	
	ICDAS 4	0	8 (26.7)	18 (60)	4 (13.3)	
	ICDAS 5	0	2 (22.2)	6 (66.7)	1 (11.1)	
	ICDAS 6	0	11 (33.3)	22 (66.7)	0	
ICDAS LL molar caries (right digit)	Intact	40 (39.2)	5 (4.9)	28 (27.5)	29 (28.4)	< 0.000001
	ICDAS 1	10 (20.4)	9 (18.4)	17 (34.7)	13 (26.5)	
	ICDAS 2	0	12 (30.8)	16 (41)	11 (28.2)	
	ICDAS 3	0	8 (66.7)	4 (33.3)	0	
	ICDAS 4	0	10 (27)	21 (56.8)	6 (16.2)	
	ICDAS 5	0	2 (28.6)	5 (71.4)	0	
	ICDAS 6	0	14 (41.2)	19 (55.9)	1 (2.9)	

*Note:* The *p*‐values are calculated using the chi‐square test.

Abbreviations: L (first letter), lower; L (second letter), left; R, right; U, upper.

### 3.4 OHI‐S

There were significant associations between “treatment types” with OHI‐S components (chi‐square, Table 6). The Kruskal–Wallis test indicated a significant difference among the OHI‐S scores in the 4 groups (*p* = 0.000, Figure S5). There were 4 significant post hoc pairwise comparisons (Bonferroni‐adjusted *p‐*values ≤ 0.009) and 2 insignificant ones: Office‎–‎Anesthesia (*p* = 0.773) and Office‎–‎No treatment (*p* = 0.225). No significant difference was detected between boys and girls (Mann–Whitney, *p* = 0.182, Figure S6).

**Table 6:** Contingency tables showing the net distributions (and percentages) of OHI‐S scores in different teeth across different treatment groups. The last item is calculated by first taking the average of OHI‐S of all teeth and then applying the thresholds of 1, 2, and 3 for good and average cases (there was no case with a poor OHI‐S index).

		Treatment groups (%)				
Parameter	Score	Healthy	No treatment	Office	Anesthesia	*p*
OHI‐S UR molar	0	14 (51.9)	3 (11.1)	6 (22.2)	4 (14.8)	0.000007
	1	27 (21.1)	22 (17.2)	46 (35.9)	33 (25.8)	
	2	7 (7.4)	23 (24.5)	46 (48.9)	18 (19.1)	
	3	2 (6.5)	12 (38.7)	12 (38.7)	5 (16.1)	
OHI‐S UL molar	0	18 (58.1)	2 (6.5)	6 (19.4)	5 (16.1)	< 0.000001
	1	25 (19.7)	26 (20.5)	45 (35.4)	31 (24.4)	
	2	5 (5.4)	15 (16.3)	51 (55.4)	21 (22.8)	
	3	2 (6.7)	17 (56.7)	8 (26.7)	3 (10)	
OHI‐S LR molar	0	13 (40.6)	3 (9.4)	9 (28.1)	7 (21.9)	0.004
	1	35 (18)	42 (21.6)	81 (41.8)	36 (18.6)	
	2	1 (2)	14 (28.6)	18 (36.7)	16 (32.7)	
	3	1 (20)	1 (20)	2 (40)	1 (20)	
OHI‐S LL molar	0	16 (50)	1 (3.1)	7 (21.9)	8 (25)	0.000002
	1	31 (16.2)	40 (20.9)	84 (44)	36 (18.8)	
	2	3 (6)	16 (32)	15 (30)	16 (32)	
	3	0	3 (42.9)	4 (57.1)	0	
OHI‐S UL central incisor	0	40 (24.5)	23 (14.1)	55 (33.7)	45 (27.6)	0.0002
	1	9 (10)	28 (31.1)	40 (44.4)	13 (14.4)	
	2	1 (3.8)	9 (34.6)	14 (53.8)	2 (7.7)	
	3	0	0	1 (100)	0	
OHI‐S LR central incisor	0	41 (20.4)	31 (15.4)	84 (41.8)	45 (22.4)	0.003
	1	9 (13.8)	20 (30.8)	23 (35.4)	13 (20)	
	2	0	8 (66.7)	2 (16.7)	2 (16.7)	
	3	0	1 (50)	1 (50)	0	
OHI‐S – all surfaces	Good	46 (22.3)	34 (16.5)	82 (39.8)	44 (21.4)	0.0005
	Average	4 (5.4)	26 (35.1)	28 (37.8)	16 (21.6)	

*Note:* The *p‐*values are calculated using the chi‐square test.

Abbreviations: L (first letter), lower; L (second letter), Left; R, right; U, upper.

## 4 Discussion

In our study, dmft values of primary teeth in different groups showed a very high value compared to the WHO goals of reaching dmft (≥ 3) in children (Organization 2013; Petersen 2004). Of course, these are justifiable by the fact that this study was not an epidemiological study and recruited subjects based on study goals.

Like any other disease, dental caries formation depends on the balance or imbalance between various factors. These include (1) invasive factors that cause the initial lesion, (2) acquired factors that change the resistance and susceptibility of tooth enamel, (3) modulating factors that exist in the environment adjacent to the teeth (i.e., plaque and saliva), and (4) several risk factors like sweet supplements (Mansur 2020; Butera et al. 2022; Thang Le et al. 2021). The occurrence of caries at a young age is a strong and perhaps even the best predictive factor for caries in the future and in adulthood (Arsang Jang et al. 2015; Powell 1998; Tellez et al. 2013). According to Gray et al., the presence of three or more carious primary molar teeth at the age of 5 years is the best predictive factor for the development of caries in the first permanent molars at the age of 7 years (Gray et al. 1991). These call for early intervention and routine follow‐up, especially in high‐risk children.

In addition to causing pain, infection, and reducing the quality of life of the child, primary tooth caries might also cause permanent tooth caries. In the study of Tellez et al. (2013), it is also stated that previous primary tooth caries are the best predictors for subsequent permanent tooth caries compared to other factors. This shows the importance of primary tooth decay in the health of permanent teeth. Also, in the study by Skeie et al. (2006), a statistically significant relationship was observed between the decay of deciduous teeth at the age of 5 years and permanent teeth at the age of 10 years. Mejàre and Stenlund (2000) considered this risk factor to be strongly related to caries formation in permanent teeth. According to Lin et al. (2021), caries of deciduous molars at the age of 3–5 years was a useful clinical factor in predicting dentinal caries on the surfaces of the first permanent molar teeth in the next 5 years. As well, Gray et al. (1991) concluded that the presence of 3 or 6 decayed primary teeth can be the best predictor for the development of permanent molar tooth decay at the age of 7 years. Moreover, Tagliaferro et al. (2006) reported that children with caries in deciduous teeth at 7 years of age were 2–3 times more likely to develop caries in their permanent teeth. According to them, no caries in milk teeth is a protective factor against caries in permanent teeth in the future. Contrary to all these studies, a study (Silva et al. 2014) concluded that premature caries in children might not be related to caries of permanent teeth, and caries alone could not be the main predictive factor.

In the present study, the ICDAS criterion was used to determine the status of all 4 permanent first molars, which is a WHO‐approved criterion. The highest ICDAS scores corresponded to patients who were untreated or under routine dental treatment; code zero was the most frequent in the healthy or general anesthesia groups. Also, enamel decays were more prevalent than dentine decays in the present sample. The highest rate of dental caries was observed in the children who were treated in the office, and then in the group without treatment. These results might be due to the fact that the treatment under anesthesia can reduce the bacterial load and microorganisms in the mouth at once due to the one‐session treatment. Moreover, due to the treatment under general anesthesia, it might be more likely that parents be considerably motivated to carefully attend to their children's oral hygiene and regular check‐ups. Of course, these are authors' suggestions that need to be verified in future studies. In this study, most patients under anesthesia attended again for routine fluoride therapy and seemed to be more enthusiastic about hygiene. Nevertheless, this was not the case for patients treated in the office, for whom the results were similar to the untreated group (with more serious permanent tooth caries). This might be due to factors such as the intervals between the treatment sessions and the lack of reduction of the bacterial load in a short time, as well as the parents caring less about their children's oral hygiene. Also, it is noteworthy that usually children who suffer from early caries at a young age need to be treated under general anesthesia. This means that the interval between the treatment of decayed baby teeth and the eruption of permanent teeth is usually longer. As a result, a longer period of time is available for the health of primary teeth and the reduction of bacterial load, and the improvement of hygiene before the eruption of permanent teeth. On the other hand, children who were treated in the office were usually older, because older children could be able to cooperate over multiple sessions, which is needed for office treatment. Therefore, the time interval between the treatment of their primary teeth and the eruption of permanent teeth was shorter. In addition, possibly due to their older age, their parents' supervision over their oral hygiene might have decreased, and they might have entrusted the brushing of their teeth to the children, which could cause a decrease in hygiene maintenance. It should be noted that all the above speculations need future verification by evidence‐based research. Lin et al. (2021) observed a result contrary to the present research, i.e., patients who were treated under general anesthesia showed optimal oral hygiene control only in the first 6 months after anesthesia (perhaps due to the high motivation of parents to supervise oral hygiene [although this needs verification by future research]); nevertheless, after that, the children's eating habits returned to the previous state and again caused decay of the permanent first molar teeth. On the contrary, in the study of Songur et al. (2019) (who examined treatment under general anesthesia and treatment under normal conditions), ICDAS 0 was more common in children treated under anesthesia than in other groups, and ICDAS code 6 (deep dentin caries) in children treated under general anesthesia was zero. On the other hand, code 6 (severe decays) was prevalent in the office treatment group, similar to those who were not treated. Also, in the study of Songur et al. (2019), similar to our study, the caries rate of the permanent first molar was higher in the group treated in the office than in the group treated under general anesthesia. But unlike our study, in their research, the group without treatment had the lowest caries rate in the permanent first molar; this could be because the group without treatment in their study probably had superficial caries without pain and infection, not requiring the parents to take any action to treat. Or maybe children with early caries, regardless of whether they are treated or not, are considered among high‐risk people for developing early caries in their permanent teeth (Songur et al. 2019).

In the present study, a significant relationship was seen between the ICDAS codes that were related to dental caries (indicating the severity of caries) and dmft. In other studies, dmft of primary teeth was moderately and linearly correlated to DMFT of permanent molars (Asgari and Ghanea 2017). Also, according to another study, there was a significant relationship between dmft and DMFT (Fallahzadeh and Hasanpour 2009). Also, 83% of children who did not have primary dentition caries remained without caries until the age of 12 (Asgari and Ghanea 2017; Li and Wang 2002), which is similar to the result of our study that ICDAS codes were only codes 0 and 1 in people with healthy primary teeth. This suggests that all primary tooth caries must be treated, and the earlier they are treated, the better. Moreover, it is suggested to discuss the microbiology of children's mouths before and after treatment in future studies.

We used the OHI‐S index, which is obtained from the sum of the plaque index and the calculus index, to evaluate the oral health status. The results of the present study showed that OHI‐S was different between some groups but not between the group treated in the office and the general anesthesia group. In the study by Songur et al. (2019), there was no difference between OHI‐S of different groups. But other studies (Rusmali et al. 2023; Hidayati et al. 2022; Doley et al. 2022) showed a direct relationship between OHI‐S and caries rate. In our study, there was also a significant association between OHI‐S and tooth decay according to ICDAS. In the study of Lin et al. (2021), children's oral hygiene improved after treatment under anesthesia, possibly due to improved motivation posttreatment – of course, this is merely the authors' opinion, needing verification by future research. However, the improvement in the oral condition only remains high for 6 months after treatment, and then gradually decreases. Therefore, a person's susceptibility to decay remains high because the behaviors that cause decay in a person do not change. Treatment under general anesthesia may improve the quality of life of children and their health because (1) dental treatment under general anesthesia is used in children with poor cooperation, (2) all teeth are treated in one session, and (3) this treatment reduces anxiety in follow‐up sessions and hence the child's cooperation gradually increases (Novaes et al. 2017; Gupta and Raghu 2021). Also, parents of children treated under general anesthesia may attach great importance to follow‐up sessions and fluoride varnish (Razeghi et al. 2020).

This non‐randomized clinical trial was limited by some factors. Ethical considerations disallow some studies to be conducted as randomized clinical trials because of contraindications or avoidable adverse effects of some treatments, or even when some treatments are not optimally effective for a particular group. In these situations, it is not possible to assign random treatments to patients; instead, the patients must be treated with the clinically recommended treatment. When such limitations are present, non‐randomized trials are the best available option. Nevertheless, they do not have the luxury of a balanced grouping. And this is by design, i.e., done intentionally and inevitably. In other words, expecting the presence of randomization and similar baseline values in such a study is irrelevant, because they are not even possible. Moreover, in non‐randomized trials, blinding is not an option, and this as well might introduce some biases. Still, the inclusion of both negative and positive control groups allowed us to rule out important confounding factors. Not to mention that the size of this trial was augmented to improve reliability. Another limitation is the significant difference between the size of the in‐office group versus the other 3 groups. Imbalances in group sizes might introduce some biases. Nevertheless, there is a trade‐off between the advantages of a larger sample size versus the disadvantages of imbalances in group sizes. We preferred to improve reliability and generalizability by enrolling as many patients as could, instead of stopping sampling after a size of about 60 children (similar to other groups). Moreover, it should be noted that all groups surpassed the minimum required size by far. Another important point to consider is the grouping criteria; in this study, grouping was not done based on the authors' preferences; grouping was done merely based on children's conditions, which were out of the authors' control. In other words, the authors could not manipulate grouping based on any potential subjective preferences and potential outcomes. Therefore, the possibility of selection bias was low. Finally, if it was possible to enroll more children with conditions of the 3 other groups, we would do it. Nevertheless, those children were rarer than routine pediatric patients. This practical limitation was the cause of this imbalance, not any negligence on the authors' part. Future studies should use more groups with larger samples and more balanced group sizes to achieve more reliable results with better control over the confounders.

## 5 Conclusions

Based on the results of this study, it can be concluded that:

1. Although children who underwent treatment with general anesthesia had the worst dmft scores, treatment under general anesthesia considerably reduced caries of their permanent first molars (as indicated by ICDAS scores) and oral hygiene (as indicated by OHI‐S) compared to children who were treated routinely in the office.
2. All correlations among primary caries (dmft), permanent molar caries (ICDAS), and oral hygiene (OHI‐S) were significant. This implies the link between oral hygiene and the caries rate of primary dentition as a decisive predictor of caries formation in subsequent permanent molars. This highlights the need for early intervention and routine follow‐up, especially in high‐risk children.
3. Age or sex were not associated with deciduous caries and dmft, permanent molar caries and ICDAS, or oral hygiene or OHI‐S scores.
4. Different treatment groups showed different primary tooth caries rates (dmft scores), different permanent molar caries rates (ICDAS scores), and, to some extent, different oral hygiene rates (OHI‐S scores).

It should be noted that despite the superior efficacy of one‐session treatment under general anesthesia, the treatment of choice should always be the in‐office routine treatment unless it is not possible. This is because treatment under general anesthesia is invasive and should be reserved only for cases that cannot be treated properly at the office.

## Author Contributions

Shirin Taravati searched the literature, conceived the study, performed the treatments, interpreted and discussed the findings, mentored the thesis, and critically reviewed the paper. Sahar Tehrani searched the literature, collected the data, interpreted the findings, and wrote the thesis. Masoumeh Khataminia searched the literature, mentored the thesis, interpreted the findings, and critically reviewed the thesis. Vahid Rakhshan searched the literature, performed the statistical analyses, interpreted the findings, ‎drafted the manuscript, and prepared figures and tables. All authors reviewed the final version and approved it for submission to this journal.

## Ethics Statement

This prospective non‐randomized clinical trial was performed in 2023–2024 in Ahvaz, Iran. The protocol and its ethics were approved by the Medical Ethics Committee of Ahvaz Jundishapur University of Medical Sciences, Ahvaz, Iran (ethical code: IR.AJUMS. REC.1402.309). All methods were performed in accordance with the relevant guidelines and regulations (including the Declaration of Helsinki); all experimental protocols were approved by the Institutional Review Board of Ahvaz Jundishapur University of Medical Sciences, Ahvaz, Iran.

## Consent

Written informed consent was obtained from all parents. The data were confidential.

## Conflicts of Interest

The authors declare no conflicts of interest.

## Supporting information


**Supplementary Figure 1:** Box plots showing descriptive statistics for dmft in the 4 study groups. **Supplementary Figure 2:** Histograms of dmft scores in boys and girls. The difference between the sexes was not significant. **Supplementary Figure 3:** Box plots for the average ICDAS scores for caries status of 4 molars. **Supplementary Figure 4:** Histograms of average ICDAS scores for caries status in boys and girls. **Supplementary Figure 5:** Box plots showing descriptive statistics for OHI‐S index in the 4 study groups. **Supplementary Figure 6:** Histograms of dmft scores in boys and girls. **Supplementary Table 1:** Description of the indices used in this study.

## Data Availability

The data that support the findings of this study are available from the corresponding author upon reasonable request.
