# Supplementary material for: Effects of Single‐Session Treatment of Primary Teeth Under General Anesthesia Versus At‐Office Multi‐session Treatment on Permanent Molar Caries Status: Nonrandomized Clinical Trial
Source: Clin Exp Dent Res. 2025 Aug 28;11(5):e70187. doi: 10.1002/cre2.70187 (PMC12392421; doi:10.1002/cre2.70187)
Supplement: Supplementary file 1 — Supplementary Figure 1: Box plots showing descriptive statistics for dmft in the 4 study groups. Supplementary Figure 2: Histograms of dmft scores in boys and girls. The difference between the sexes was not significant. Supplementary Figure 3: Box plots for the average ICDAS scores for caries status of 4 molars. Supplementary Figure 4: Histograms of average ICDAS scores for caries status in boys and girls. Supplementary Figure 5: Box plots showing descriptive statistics for OHI‐S index in the 4 study groups. Supplementary Figure 6: Histograms of dmft scores in boys and girls. Supplementary Table 1: Description of the indices used in this study. [file CRE2-11-e70187-s001.docx]

**Supplementary Table 1.** Description of the indices used in this study.

| Index | Description |
| --- | --- |
| dmft | In this index, d (decay) is the total number of primary teeth that are carious, m (missing) is the total number of deciduous teeth that are lost due to decay, and f (filling) represents the number of restored primary teeth. |
| OHI-S | The OHI-S index is obtained from the sum of the calculus index (CI) and the plaque index (debris index) DI. For this index, we examined six dental surfaces: the buccal surface of the upper right first molar, the labial surface of the upper right central incisor, the buccal surface of the upper left first molar, the lingual surface of the lower left first molar, the labial surface Lower left central incisor, and lower right first molar lingual surface. The CI and DI are measured for each of these surfaces, the numbers are added together and divided by the number of surfaces, which is 6; in other words, the average of the CI + DI of 6 surfaces is calculated as the OHI-S. The calculus index (CI) is evaluated as follows: a score of 0 for the absence of any calculus on the mentioned surfaces, a score of 1 for a calculus above the gums that does not cover more than one third of the tooth surface, a score of 2 for a calculus above the gums which covers more than one-third and less than two-thirds of the tooth, and a score of 3 is given for the upper gingival calculus that covers more than two-thirds of the tooth surface. The plaque index (DI) is evaluated as follows: a score of zero is for the absence of any plaque, debris, and pigment on the dental surfaces; score 1 for the presence of soft debris and external pigment on one-third of the tooth surface, score 2 for the presence of soft debris on more than one-third of the tooth surface and less than two-thirds of the tooth surface, and score 3 for the presence of debris on more than two-thirds of the tooth surface. After adding CI and DI scores and dividing the sum by 6, the value of OHI-S is obtained. An OHI-S number between 0 and 1.2 is considered appropriate, between 1.3 and 3 is considered average, and between 3.1 and 6 is considered poor. |
| ICDAS – the left digit | The numbers 0 to 8 are used for the first digit that shows the restoration status: code 0: healthy tooth, code 1: partial sealant, code 2: complete sealant, code 3: composite filling, code 4: amalgam, code 5: stainless steel crown, code 6: other crowns, code 7: fallen/broken filling, code 8: temporary filling. |
| ICDAS – the right digit | This right digit shows the state of tooth decay in the form of a code between 0 and 6: The number 0 is reserved for a healthy tooth, which does not show any signs of translucency alteration (when the tooth is moist and when the tooth is dried for 5 seconds with an air blower). The number 1 is assigned to the first optical change in the enamel, and in this case, when we dry the tooth with air for 5 seconds, a decrease in translucency and an increase in opacities are seen. The number 2 is a state in which the translucency change is seen in both the dry and wet states of the tooth, and this group is called "obvious optical change" in the enamel. The number 3 is a condition where the enamel is disrupted and the integrity of the enamel is lost, but the cavity is at the level of the enamel and has not entered the dentin. The number 4 indicates the appearance of a dark shade of the underlying dentin, which can be accompanied by diseased enamel or not. The number 5 indicates a clear cavity in a way that the bottom of the cavity has entered the dentin. The number 6 shows extensive caries in the dentin region, which has created a wide and deep cavity, where both the bottom of the cavity and its walls are in the dentin. The last two scores are indications for root canal therapy. |
| ICDAS – for missing teeth | Aside from the above ICDAS numbers, there are 4 additional 2-digit codes that are reserved for missing teeth: 96 = cannot be examined, 97 = missing due to caries, 98 = missing due to other reasons, and 99 = impacted teeth. If a surface has several carious lesions, the highest code would be assigned to that surface. |


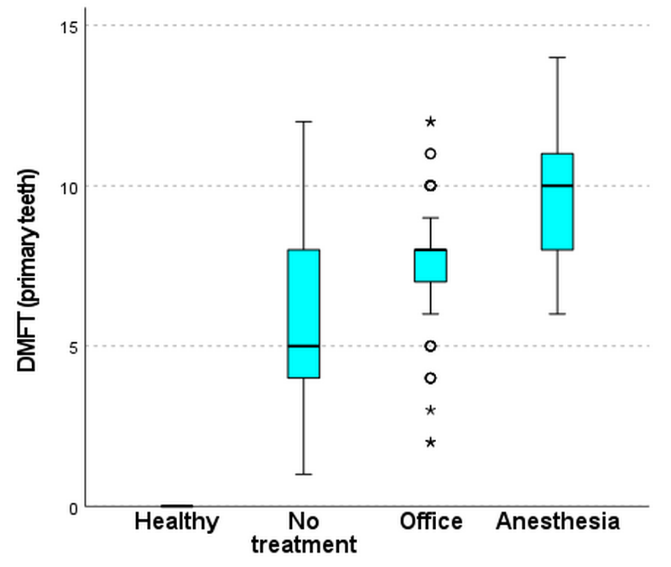


**Supplementary Figure 1.** Box plots showing descriptive statistics for dmft in the 4 study groups. All pairwise comparisons were statistically significant. Circles denote mild outliers that fall between 1.5 and 3 times the interquartile range (IQR) away from the edge of the box, while asterisks represent extreme outliers that fall beyond 3 times the IQR.


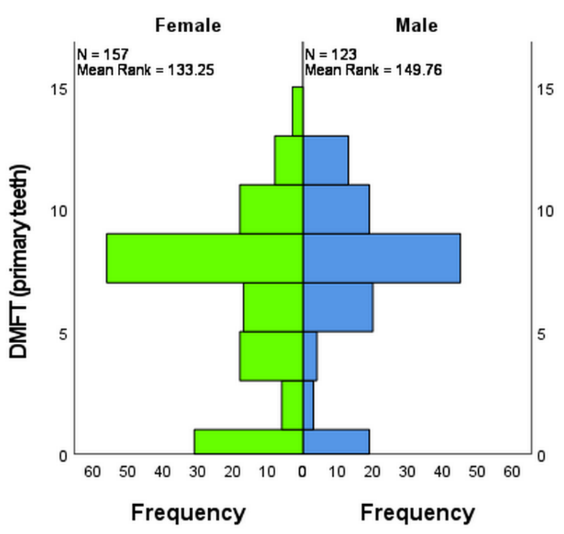


**Supplementary Figure 2.** Histograms of dmft scores in boys and girls. The difference between the sexes was not significant.


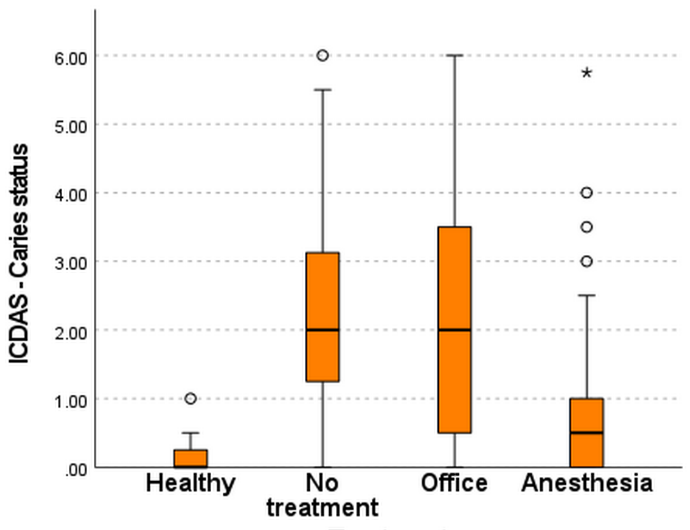


**Supplementary Figure 3.** Box plots for the average ICDAS scores for caries status of 4 molars. Except for the comparison between office treatment versus positive control group, the rest were statistically significant. Circles denote mild outliers while asterisks represent extreme outliers.


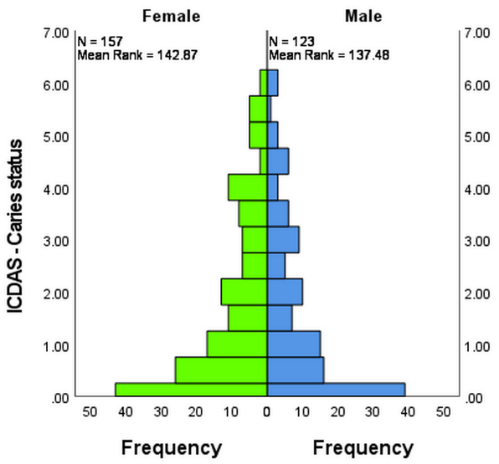


**Supplementary Figure 4.** Histograms of average ICDAS scores for caries status in boys and girls. The difference between the sexes was not significant.


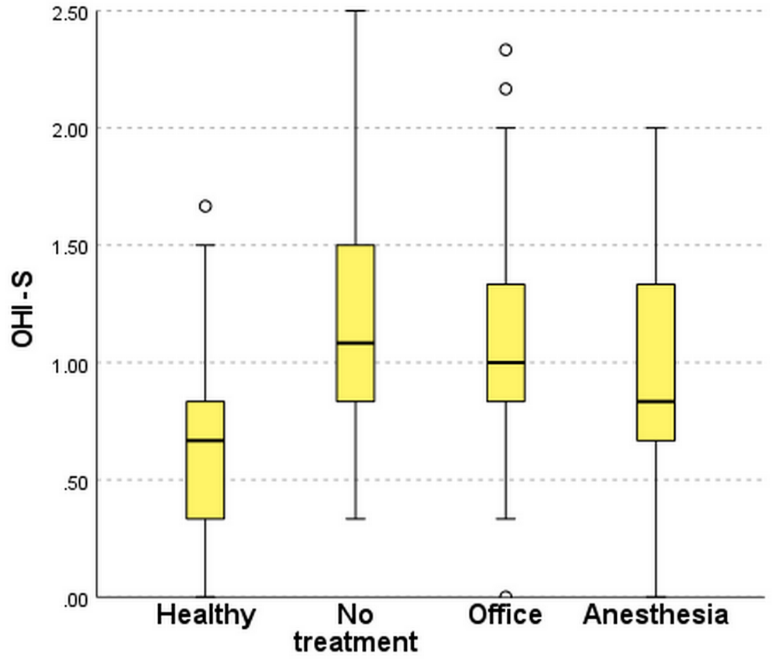


**Supplementary Figure 5.** Box plots showing descriptive statistics for OHI-S index in the 4 study groups. Except the pairwise comparisons Anesthesia-Office and Office-No treatment, the other 4 pairwise comparisons were statistically significant. Circles denote mild outliers that fall between 1.5 and 3 times IQR away from the edge of the box.


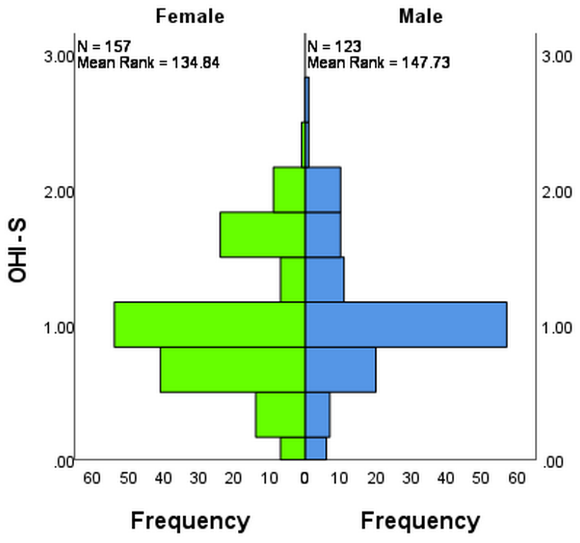


**Supplementary Figure 6.** Histograms of dmft scores in boys and girls. The difference between the sexes was not significant.
